# Dual sources of melatonin and evidence for different primary functions

**DOI:** 10.3389/fendo.2024.1414463

**Published:** 2024-05-14

**Authors:** Russel J. Reiter, Ramaswamy Sharma, Dun-Xian Tan, Luiz Gustavo de Almieda Chuffa, Danilo Grunig Humberto da Silva, Andrzej T. Slominski, Kerstin Steinbrink, Konrad Kleszczynski

**Affiliations:** ^1^ Department of Cell Systems and Anatomy, UT Health San Antonio, Long School of Medicine, San Antonio TX, United States; ^2^ Applied Biomedical Sciences, University of the Incarnate Word, School of Osteopathic Medicine, San Antonio, TX, United States; ^3^ Departamento de Biologia Estrutural e Funcional, Setor de Anatomia - Instituto de Biociências, IBB/UNESP, Botucatu, São Paulo, Brazil; ^4^ Department of Biology, Universidade Estadual Paulista (UNESP), São Paulo, Brazil; ^5^ Department of Biology, Universidade Federal de Mato Grosso Do Sul, Três Lagoas, Mato Grosso Do Sul, Brazil; ^6^ US and Pathology Laboratory Service, Department of Dermatology, University of Alabama at Birmingham, Birmingham, AL, United States; ^7^ Department of Dermatology, University of Münster, Münster, Germany

**Keywords:** extrapineal melatonin, circadian rhythms, suprachiasmatic nucleus, mitochondria, redox homeostasis, free radicals, cerebrospinal fluid, cell metabolism

## Abstract

This article discusses data showing that mammals, including humans, have two sources of melatonin that exhibit different functions. The best-known source of melatonin, herein referred to as Source #1, is the pineal gland. In this organ, melatonin production is circadian with maximal synthesis and release into the blood and cerebrospinal fluid occurring during the night. Of the total amount of melatonin produced in mammals, we speculate that less than 5% is synthesized by the pineal gland. The melatonin rhythm has the primary function of influencing the circadian clock at the level of the suprachiasmatic nucleus (the CSF melatonin) and the clockwork in all peripheral organs (the blood melatonin) via receptor-mediated actions. A second source of melatonin (Source # 2) is from multiple tissues throughout the body, probably being synthesized in the mitochondria of these cells. This constitutes the bulk of the melatonin produced in mammals and is concerned with metabolic regulation. This review emphasizes the action of melatonin from peripheral sources in determining re-dox homeostasis, but it has other critical metabolic effects as well. Extrapineal melatonin synthesis does not exhibit a circadian rhythm and it is not released into the blood but acts locally in its cell of origin and possibly in a paracrine matter on adjacent cells. The factors that control/influence melatonin synthesis at extrapineal sites are unknown. We propose that the concentration of melatonin in these cells is determined by the subcellular redox state and that melatonin synthesis may be inducible under stressful conditions as in plant cells.

## 1 Introduction

During their early investigative history, the pineal gland/organ and associated epithalamic structures were morphologically described in detail in many vertebrate species. A significant portion of these publications reported that the outgrowths of the posterodorsal thalamus, i.e., the pineal and the frontal organ (also collectively known as the pineal complex in amphibians), contain photoreceptors much like those in the lateral eyes suggesting they respond directly to light penetrating the area; at that time, the pineal was often referred to as the third eye (1). Further evidence such as the presence of a cartilaginous transparent plate enclosing the occipital fontanel, which allowed the easy penetration of light into the epithalamus, indicated the same as did electrical activity recordings from the light stimulated pineal in species such as the teleost*, Salmo irideus* (2). With the discovery of a genuine dark-dependent specific secretory product (melatonin) in the mammalian pineal gland in the late 1950s, the investigative landscape of the gland dramatically and rapidly changed with developments in the field currently occurring at an almost exponential rate (3).

It is now apparent that melatonin is not exclusively of pineal origin. There are two major endogenous sources of this molecule, herein referred to as Source # 1 (pineal melatonin) and Source # 2 (melatonin produced in extrapineal tissues). These melatonin pools are differentially regulated and, based on current evidence, also have different primary functions. These are concepts elaborated in the current review.

## 2 Pineal melatonin (Source # 1)

### 2.1 The backstory

N-acetyl-5-methoxytryptamine, commonly known as melatonin, was isolated and chemically identified in bovine pineal tissue (4, 5). Prior to that, the pineal gland was considered to be evolutionarily vestigial. The impetus for this study was actually published 50 years earlier when it was reported that feeding frog larvae (tadpoles) minced bovine pineal glands caused their skin to lighten dramatically due to the aggregation of the melanin pigment around the nucleus of skin chromatophores (6). Lerner and colleagues (5), since they were dermatologists, surmised that the isolated factor of pineal origin might be useful in treating the human skin disorder known as vitiligo. Unfortunately, after its isolation and when tested in humans, melatonin did not appreciably alter the abnormal skin pigmentation in human melanocytes (7). Shortly after its discovery, the biosynthetic pathway of melatonin from serotonin was identified; thus, it was shown that serotonin is first N-acetylated to form N-acetylserotonin which is then O-methylated resulting in the formation of melatonin (8, 9).

Almost concurrent with the identification of the biosynthetic pathway for melatonin from serotonin, investigators initiated studies related to the effects of the light:dark cycle on pineal melatonin synthesis. These investigations were stimulated by earlier observations showing that pinealocyte morphology changed due to alterations in the photoperiod to which animals were exposed (10, 11). Thus, Wurtman and co-workers (12) exposed rats to continual darkness for 6 days and reported a striking rise in the activity of the melatonin synthesizing enzyme hydroxyindole-O-methyltransferase (HIOMT) (now called serotonin N-acetyltransferase/ASMT) and surmised that pineal melatonin production also was elevated during the dark. While they were correct in their assumption that pineal melatonin synthesis is elevated during darkness, subsequent studies have not shown that HIOMT/ASMT rises appreciably during the daily dark period. Rather, N-acetyltransferase (NAT), which controls the conversion of serotonin to N-acetylserotonin, exhibits a very large nighttime rise in activity and is rate-limiting enzyme in pineal melatonin production (13).

**Figure 1:**
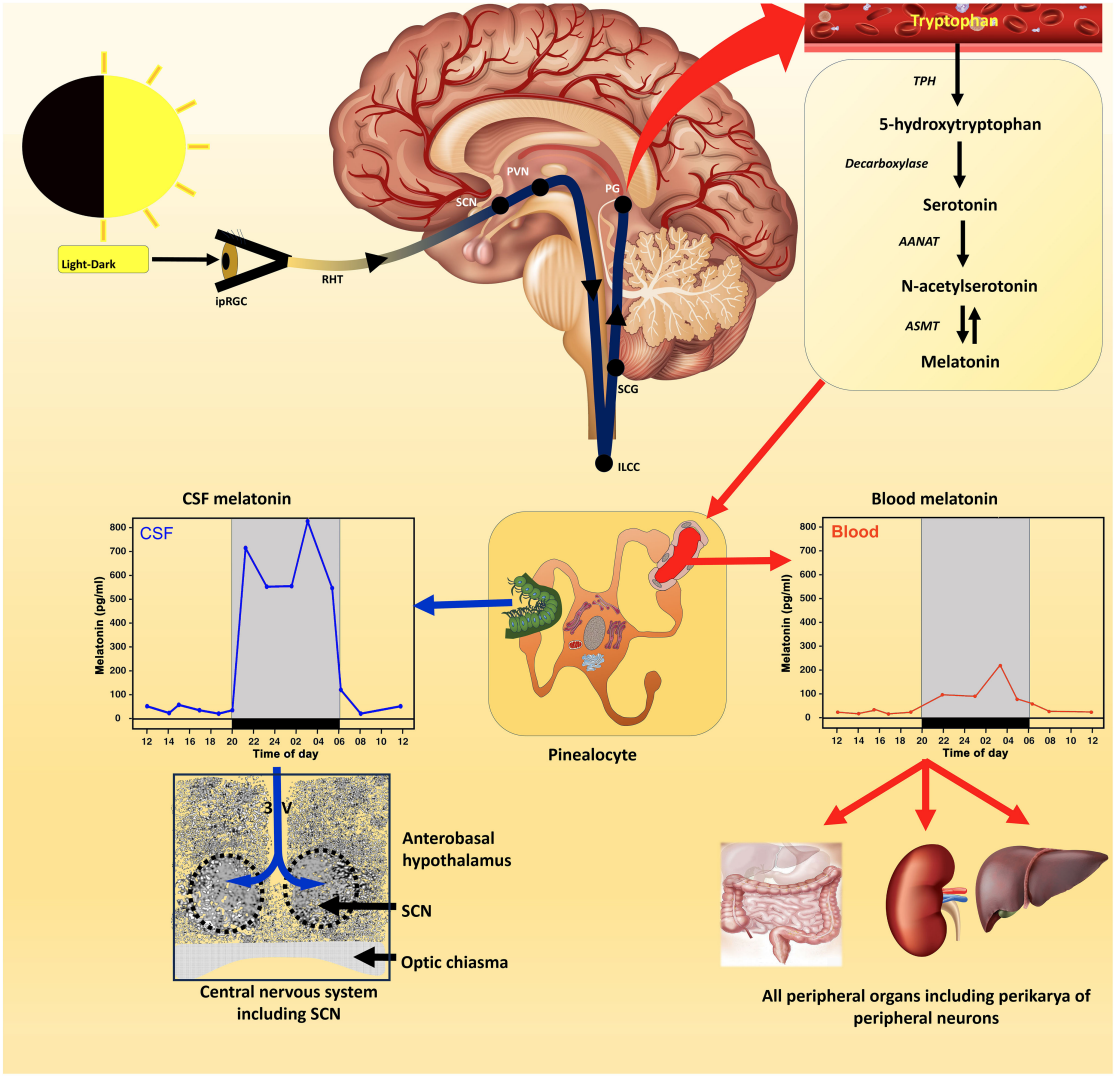
The control of melatonin synthesis in the pineal gland (Source # 1) of vertebrates, especially mammals, has been thoroughly investigated from the level of the eyes to the release of melatonin from the gland (top panel); numerous excellent articles and reviews have discussed these subjects which the reader can consult for details. Conventionally, it was thought that the primary route of melatonin secretion was into the perfuse capillary bed in the pineal with nocturnal blood levels exhibiting a measurable rise (middle panel); blood values are typically in the pg/mL range in this fluid. Melatonin is also released into the third ventricular cerebrospinal fluid (CSF) with the rhythm in this fluid exhibiting a much greater amplitude than in the blood. Melatonin diffuses quickly throughout the ventricles and at the base of the third ventricle melatonin presumably has easy access to the master circadian oscillator (suprachiasmatic nucleus; SCN) either due to simple diffusion or transfer via tanycytes. This feedback effect of melatonin on the circadian clock, which is receptor mediated, has an important role in synchronizing circadian processes within the SCN helping to ensure well-regulated 24-hour rhythms throughout the body, e.g., sleep-wake cycle (a primary function of Source # 1 melatonin). The duration of the nocturnal rise in melatonin which is dependent on seasonally changing day/night length also mediates circannual rhythms. The SCN influences circadian rhythms via the visceral/involuntary nervous system (the autonomic nervous system) which innervates smooth and cardiac muscle and many exocrine and endocrine glands. Since all cells are assumed to possess clock genes, the circadian blood melatonin cycle is believed to influence their expression including cells not directly innervated by the autonomic nervous system. In advanced age, the SCN/pineal/melatonin axis deteriorates leading to weaken circadian rhythms which negatively impact disease incidence and general health.

In **Figure 1**, the enzyme regulating the conversion of serotonin to N-acetylserotonin is listed as AANAT (arylalkylamine N-acetyltransferase). The acronym, NAT, is the more broad-spectrum arylamine N-acetyltransferase that also acetylates serotonin, which allows for the production of melatonin in species that are deficient in AANAT and incorrectly defined as melatonin knock out (14); this enzyme allows for melatonin production in peripheral organs as described by Slominski and colleagues (15). In addition, acetylation of serotonin by the alternative to AANAT was described not only in C57BL/6 mouse but also in humans, rats and hamsters (16–18).

Quay was the first to document that pineal melatonin synthesis was cyclic and that its production was clearly higher at night than during the day (19). Moreover, it was quickly shown that the function of the pineal gland (20) as well as its biosynthetic activity (12) are related to the light:dark cycle perceived by the lateral eyes rather than being dependent on direct photostimulation as had been reported in some amphibians and reptiles (21). This dependency was not unexpected considering the already-described definition of the neural connections between the visual system and the rat pineal gland (22). The circadian rhythm in pineal melatonin synthesis and secretion has now been confirmed in dozens of mammalian species, including in the human (23) and is considered axiomatic (**Figure 1**).

### 2.2 Functions of pineal-derived melatonin (Source # 1)

While Lerner and Nordlund failed to show that oral melatonin altered pigment distribution in human melanocytes as with amphibian larvae (7), interest quickly shifted to pineal (melatonin)/reproductive interactions. These studies were performed since even prior to the discovery of melatonin, books authored by Kitay and Altschule (24) and Thieblot and LeBars (25), had hinted at the possibility that pineal function may impact reproductive physiology. Different approaches were taken for these investigations; Wurtman et al. (26) treated rats with melatonin, maintained them under long photoperiods and thereafter examined pubertal development and the estrous cycle, based on the time of vaginal opening and on daily vaginal smears. The results were ambiguous with very minor changes seen in pubertal onset and estrous cycle perturbations. The choice of the rat for this study was not optimal, since its reproductive system is essentially insensitive to pineal removal or to melatonin given that this highly inbred species is not a seasonal breeder (27). By comparison, pineal removal (with the loss of the circadian melatonin rhythm) prevented the dramatic reproductive collapse that occurs in the photosensitive Syrian hamster (*Mesocricetus auratus*) kept under short, winter-type photoperiods; this effect was observed in both male and female hamsters (28, 29). These were the first results to unequivocally document an important role for the pineal gland and melatonin on any aspect of physiology in a mammal. Furthermore, these findings, along with others, led to the now well-established theory that seasonal fluctuations in reproductive capability in photosensitve species are a result of the changing duration of the elevated nocturnal pineal melatonin secretion, which is determined by the annual fluctuations in night length (30, 31). Thus, the melatonin rhythm provides both clock and calendar information (32).

One feature that has complicated the identification of the mechanisms by which the changing melatonin signal regulates annual reproductive changes in seasonally breeding mammals is the fact that in the Syrian hamster (a long day breeder) the reproductive organs are atrophic in the winter which prevents mating, pregnancy and delivery of the young (30). Conversely, in seasonally breeding sheep (a short-day breeder) just the opposite occurs; thus, they are reproductively competent in the short days of the winter but not in the summer (33). Thus, the long duration daily melatonin rise, typically of the short days of the winter, can either inhibit or promote reproductive capability. This suggests that melatonin is neither pro- nor anti-gonadotrophic; rather it is a passive signal of night length which provides time of year information (calendar) (32) with the use of the message being species dependent. A recent review (34) summarizes some of the hypothalamic mechanisms by which melatonin mediates seasonal reproductive changes.

Based on the rhythmic secretion of melatonin from the pineal, the circadian actions of the molecule were quickly investigated and thousands of reports related to this subject have been published (35). This important research has led to advances in our understanding of the function of the master circadian pacemaker, i.e., the suprachiasmatic nucleus (SCN) (36), which melatonin helps to entrain and to the novel mechanisms of photoreception involved in the regulation of physiological rhythms generally and the pineal melatonin cycle specifically (**Figure 1**) (37).

The melatonin/circadian rhythm field is massive with many clinical applications already having been proposed or established (38). Many of these studies relate to melatonin’s ability to promote sleep (39). It seems likely that there is no system in the organism, either normal or pathological, that avoids the influence of the SCN or the melatonin cycle (40, 41). Clock genes related to these actions are found in essentially every cell (42, 43).

Since the circadian melatonin cycle has been identified in the blood of every mammalian species where it has been examined (with the possible exception of some mice which genetically lack the enzymatic machinery to synthesize melatonin in the pineal gland), it was a reasonable assumption that the synchronizing actions of melatonin on the SCN is a result of the variation in day:night blood melatonin levels (44). Besides releasing melatonin into the circulation in small amounts (usually levels are in the range of pg/mL), melatonin is also discharged directly into the cerebrospinal fluid (CSF) of the third ventricle and is likely an important primary secretory pathway since the nocturnal elevation in this fluid is roughly an order of magnitude greater than that in the blood (**Figure 1**) (45). Since melatonin in the third ventricular CSF would have easy access to the nearby SCN, it has been suggested that the greater amplitude CSF melatonin cycle is actually responsible for its synchronizing effect at the level of the SCN (46). Under any circumstances, the relationship between melatonin and control of circadian rhythms, and thereby also circannual cycles by the SCN, is indisputable and are major functions of pineal-derived melatonin (Source #1) (30, 47, 48).

While the circadian melatonin rhythm in the CSF may synchronize the activity of the SCN, it seems likely that the blood melatonin cycle regulates the clock genes in peripheral cells (**Figure 1**) (49) along with neural information that arrives from the SCN via the autonomic nervous system innervation. The ability of the melatonin cycle to adjust circadian biology is also consistent with the observations that the diminished melatonin levels in the aged are associated with generalized chronodisruption (35). It is not known, however, whether the deteriorating melatonin values in the aged interrupt normal SCN physiology or whether the faltering SCN function causes the drop in melatonin secretion since these actions are mutually dependent. Seemingly the major functions of the cyclic production of melatonin by the pineal gland of vertebrates relate to circadian biology.

Surgical removal of the pineal gland in mammals is usually associated with essentially an absence of blood melatonin levels with no discernible rhythm. Pinealectomy in some poikilothermic vertebrates, however, does not result in a loss of the circadian blood melatonin cycle (50). Also, in these species, the melatonin rhythm may be dictated by day:night ambient temperature variations. Melatonin is associated with the pineal gland only in vertebrates since they are the only species that have this organ; still, this indoleamine is found (Source #2) throughout the animal and plant kingdoms including protists and in plants, all species that lack a pineal gland and have no equivalent homolog. There are also some vertebrates that lack a morphological discernible pineal gland, but still exhibit a light/dark-related low amplitude blood melatonin cycle, e.g., the alligator (*Alligator mississippiensis*) (51), and other members of the subclass of Archoasurian reptiles. The authors surmised that the melatonin cycle in these species is probably not involved in circadian regulation; moreover, they felt this rhythm does not originate from pineal tissue, i.e., rather being of extrapineal origin, since this structure is morphological absent. It seems more likely, however, that these species have functional pinealocytes diffusely distributed in their epithalamus that are not organized into an identifiable discrete gland.

## 3 Extrapineal melatonin (Source # 2)

### 3.1 The backstory

Within a year after the discovery of melatonin in the pineal gland (5), the same group of investigators also found melatonin in the sciatic nerves of humans and in other mammalian peripheral nerves (52). While they did not speculate on its origin since they did not examine its local synthesis, most likely they thought it was pineal-derived melatonin taken up from the blood.

Since then, melatonin has been identified in many cells not associated with the pineal gland; this is generally referred to as extrapineal melatonin (Source#2) (53, 54). The amount of melatonin found outside the pineal gland is massive in comparison with what presumably could be produced in this small neural outgrowth where its synthesis only occurs during the daily dark period. This also becomes highly relevant in animals living at extremely high latitudes where persistent darkness for long periods totally eliminates the blood melatonin rhythm, sometimes for multiple months of the year (55). If the total melatonin load in these species was derived exclusively from the pineal gland, they would be devoid of all melatonin for a significant portion of each year. Also noteworthy is that non-vertebrate species, that is, invertebrates, protists and plants, also contain melatonin, sometimes in much higher concentrations than in vertebrates (56–58). It seems unlikely that the cells of millions of species that lack any semblance of a pineal gland would synthesize melatonin, while vertebrate non-pineal cells would not do so.

**Figure 2:**
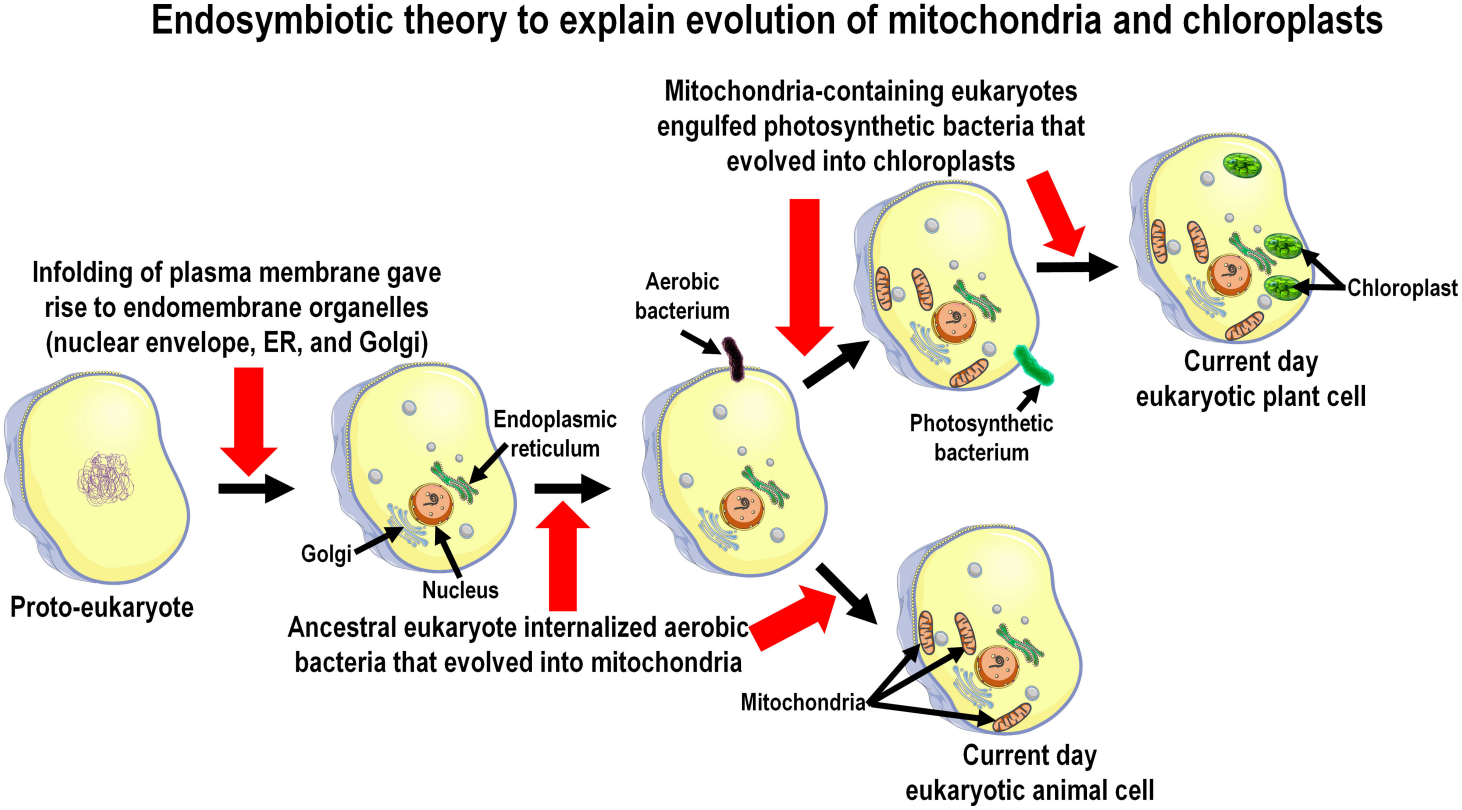
The presence of melatonin in the mitochondria and chloroplasts of present-day species is consistent with the endosymbiotic theory which explains the derivation of these organelles, which is diagrammatically summarized in this figure. Proto-prokaryotic organisms (bacteria) are estimated to have evolved perhaps as early as 3.7 to 3.5 billion years ago; over the next billion and a half years these cells developed intracellular organelles resulting in the formation of primitive eukaryotes. Melatonin synthesizing capacity in prokaryotes predictably evolved during the geologic period referred to as the Great Oxidation Event (2.5 to 2.0 billion years ago) which necessitated organisms to develop antioxidants to evade the toxicity of oxygen-based derivatives. During their evolution eukaryotes engulfed/phagocytized prokaryotes for food. Over time these engulfed organisms, i.e., either protobacteria (forming mitochondria) or photosynthesizing cyanobacteria (forming chloroplasts), developed a symbiotic relationship with their host cells. The host cells took advantage of the energy producing network and the melatonin-forming capacity of the engulfed prokaryotes and, because of these functions were beneficial, both were preserved in all eukaryotic cells that exist today.

The proposed evolution of melatonin also predicts that melatonin would be produced in many cells/organs in addition to the pineal gland (59, 60). As currently theorized, melatonin evolved 2.5 to 2.0 billion years ago (bya) in bacteria at a time when eukaryotes did not yet exist, with its initial function being that of a reactive oxygen species (ROS) scavenger (61). Its evolution may have occurred in association with the Great Oxidation Event (2.5-2.0 bya) when the Earth’s atmospheric concentrations of oxygen rose profoundly because of its release from the photosynthesizing microbes, cyanobacteria (**Figure 2**) (62, 63). Although the photosynthetic prokaryotes, cyanobacteria, did not possess chloroplasts, they had membranous photosynthesizing pigments which could capture and use solar energy for photosynthesis. Due to the toxicity of the oxygen-based derivatives (ROS), the need for protective antioxidants increased, presumably resulting in the evolution of melatonin and other ROS neutralizing or metabolizing species (64). The evolution of melatonin presumably occurred concurrently in both photosynthesizing cyanobacteria and in non-photosynthesizing bacteria, e.g., α-proteobacteria, of which multiple types share a common ancestor (65).

With the arrival of primitive eukaryotes, they internalized melatonin-producing bacteria as food or as an energy source. The phagocytized non-photosynthetic bacteria (symbionts) over eons evolved into mitochondria while the engulfed photosynthesizing bacteria became chloroplasts (known as the well-established endosymbiotic theory) of the early eukaryotes and during this process the melatonin-synthesizing capacity of the bacteria-derived organelles, both chloroplasts and mitochondria, was retained (**Figure 2**). Moreover, since all present-day eukaryotic cells (with few exceptions) contain mitochondria, chloroplasts, or both, they continue to be a source of melatonin (see below). The pineal gland, which is only found in vertebrates (which did not appear until the Cambrian explosion about 520 million years ago; mya), is a rather recently evolved organ for melatonin synthesis. Thus, melatonin likely existed for millions of years before the pineal gland even evolved. When melatonin receptors first appeared has not been thoroughly investigated but is surmised to have occurred during the Triassic Period (250 mya) (66), so the functions of melatonin (and its metabolites) prior to that time were receptor-independent, e.g., they functioned in the modulation of metabolic processes without an intervening receptor including as a direct ROS scavenger, etc. (61). Both non-receptor-mediated and receptor-dependent functions of melatonin are retained in present day vertebrate species (67).

Numerous papers documenting the presence of melatonin in invertebrates and in protists, including algae, which are not classified as plants although most algae species have the capacity of photosynthesis for energy production. The findings of these reports prompted investigations into examining the possible presence of melatonin in photosynthetic land plants. Three reports were published in 1995 by independent groups of investigators that simultaneously showed unequivocally that the plants tested contain melatonin. In these studies, multiple techniques were used to identify melatonin in the plant species examined (68–70); shortly thereafter, it was reported that the melatonin concentration in the seeds of edible plants varies widely (71). Furthermore, melatonin has been identified in all plant organs: leaves, stems, roots, flowers, etc. (72), some of which contain cells capable of photosynthesis and others that do not. Since melatonin produced by plant cells cannot be quickly circulated/transferred among plant organs, it is likely that all cells generate this indoleamine, i.e., a single plant tissue does not produce melatonin which is then distributed to other plant appendages.

Surgical removal of the pineal gland in mammals is always associated with essentially an absence of blood melatonin levels with no discernible rhythm. Melatonin is associated with the pineal gland only in vertebrates since they are the only species that have this organ; but the indoleamine is found in invertebrates including protists and in plants, all species that lack a pineal gland and have no pineal homolog.

### 3.2 The role of extrapineal melatonin (Source # 2): regulation of redox homeostasis

Studies on the actions of melatonin in relation to circadian biology continue to be intensively investigated; this action clearly involves the cyclic production and release of melatonin from the pineal gland (Source #1). More recently, other investigations have proven that the actions of melatonin far exceed those that influence clock functions. Mounting evidence in recent years supports the extensive interaction between the circadian and redox systems. Such a relationship is not surprising because most diurnal or nocturnal organisms display daily oscillations in energy intake, locomotor activity, and exposure to exogenous and internally generated oxidants (73). The transcriptional clock controls the levels of many antioxidant proteins and redox-active cofactors. Conversely, the cellular redox state has been shown to feed back to the transcriptional oscillator via redox-sensitive transcription factors and enzymes. Thus, these intrinsic clocks are thought to have co-evolved with cellular redox regulation (73, 74).

The discovery and repeated confirmation of melatonin as an oxygen-based free radical scavenger and antioxidant represents a paradigm shift (61, 75–82), especially since this applied to melatonin at both the pinealocyte level, and to extrapineal cells (Source # 2). This provides all cells additional protection against the continual bombardment and potential molecular destruction caused by ROS as well as by reactive nitrogen species (RNS) (83–85). Even though melatonin and its metabolites are highly efficient radical detoxifiers (86–88), it is apparent that the small quantity of melatonin released from the pineal gland on a nightly basis would not be sufficient to combat or neutralize the total radical free load that an entire organism generates every day. Thus, if melatonin does function as a system-wide antioxidant, sources of melatonin other than the pineal gland would be necessary. Obviously, this is even more apparent in invertebrates, protists, and plants that have no pineal gland. This problem was solved when melatonin was identified in many peripheral organs (53, 89, 90) and even more so when it is located in the mitochondria of these cells as has been shown (91).

**Figure 3:**
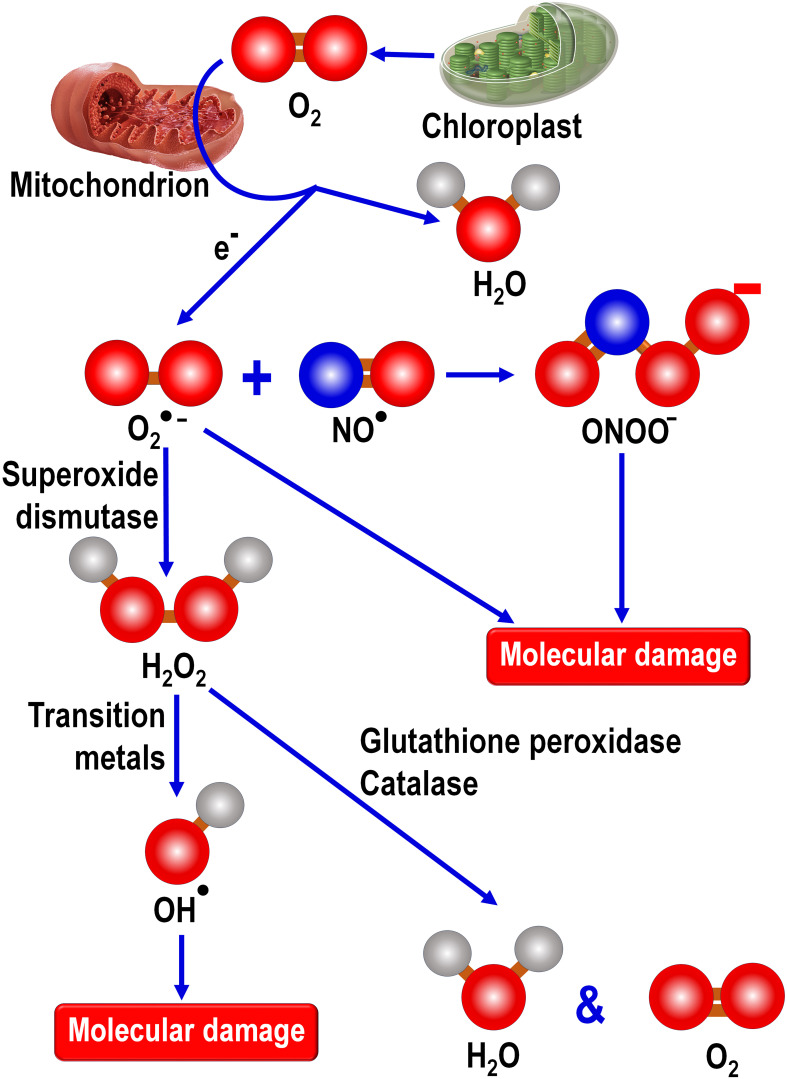
During photosynthesis by land plants, chloroplasts released oxygen as a byproduct of photosynthesis. During the Great Oxidation Event more than 2 billion years ago, atmospheric oxygen gradually rose as a consequence of oxygen released by photosynthetic prokaryotes, cyanobacteria. Early in evolution, engulfed cyanobacteria gave rise to chloroplasts in primitive photosynthesizing eukaryotes which, to this day, contribute highly significantly to atmospheric oxygen levels along with that derived from land plants. The metabolism of oxygen by mitochondria, which are present in essentially all animal and plant eukaryotes, generates the superoxide anion (O_2_^●^ **^-^**) radical which is the precursor of a number of other toxic oxygen-based and nitrogen-based products. O2^●^ **^-^** couples with nitric oxide (NO^●^) to form peroxynitrite (ONOO**^-^**), a powerful oxidizing agent. The enzymatic dismutation of O_2_^●^ **^-^** forms hydrogen peroxide (H_2_O_2_), a non-radical product that exhibits high diffusibility and which can be metabolically degraded by either glutathione peroxidase or catalase. In the presence of a transition metal, often Fe^2+^, H_2_O_2_ is converted to the highly reactive and destructive hydroxy radical (OH^●^). Cells have no means to enzymatically remove the OH^●^ and its extremely short half-life means it must be formed in the vicinity of a radical scavenger if it is to be incapacitated before it inflicts molecular damage. Thus, since OH^●^ is formed in high concentrations in mitochondria, it is important that melatonin also be situated in this organelle to be available for scavenging this reactant. As a direct free radical scavenger, melatonin reportedly neutralizes the O_2_^●^ **^-^**, H_2_O_2_, OH^●^, NO^●^ and ONOO**^-^**. Additionally, melatonin stimulates the radical metabolizing enzymes superoxide dismutase, glutathione peroxidase and catalase. It also enhances the concentration of another important antioxidant, glutathione, by stimulating the rate limiting enzyme, glutamine cysteine ligase, required for glutathione synthesis.

Not only is melatonin produced by peripheral cells, but importantly it is likely generated specifically in mitochondria (**Figure 3**) (59, 92, 93); this is critical since these organelles are major contributors to free radical generation. The presence of melatonin in these organelles is likely of special importance when there is a rapid deluge of newly produced radicals such as occurs during exposure to ionizing radiation (94), injurious chemicals such as paraquat (95), or toxic drugs such as including chemotherapies (96). During these precarious situations it is essential to have a multifaceted antioxidant in mitochondria to quickly neutralize the highly reactive ROS/RNS to avoid functional deterioration of these critically-important organelles. Moreover, if melatonin is inducible in animal cell mitochondria, as we suspect it is, it would be even more beneficial in combatting the acute free radical toxicity of hazardous processes or toxins. The likely upregulation of melatonin synthesis in stressed mitochondria is also strongly supported by abundant data showing that plant cells exhibit a rapid compensatory upregulated melatonin production when they are exposed to radical-mediated stresses resulting from multiple events, e.g., cold or hot ambient temperatures (97, 98), increased salinity (99) or chemical exposure (100), drought (101), etc. That melatonin scavenges radicals in mitochondria is well documented (102).

Locally produced melatonin also protects the intramitochondrial environment from oxidative stress since in addition to acting as a direct radical scavenger it also stimulates enzymes, i.e., superoxide dismutase 2 (SOD2) and glutathione peroxidase (GPx), that remove ROS from the mitochondrial matrix (103). The elevated SOD2 activity is achieved as a result of an upregulation of sirtuin 3 (SIRT3), one member of a family of epigenetic enzymes located primarily in the mitochondrial matrix that regulates a variety of metabolic processes (104, 105). Also, SIRT3 is upregulated by oxidative stress in both mammalian as well as in invertebrate neurons which in turn stimulates the detoxification of ROS due to its capacity to promote the activity of SOD (106). This latter observation suggests the possibility that oxidative stress is consequential in the reported compensatory rise in subcellular melatonin production, as commonly observed in plants and which has also been reported in algae (97, 107), which in turn upregulates the SIRT3/FOXO/SOD2 pathway, thereby helping to maintain oxidative homeostasis under elevated oxidative stress conditions. This would obviously be a very important function of Source # 2 melatonin in animals.

The normal nocturnal rise in pineal melatonin and its release (Source # 1) are mediated due to the interaction of postganglionic neuron norepinephrine (NE) with specific receptors on the pinealocyte membrane (108). Pineal melatonin production is not inducible; when stress-mediated circulating NE rise during the day, the nerve endings in the pineal gland reportedly act as a sink to sequester high blood NE levels (109, 110). Thus, pineal melatonin synthesis is not elevated as a result of systemic stress which causes the discharge of catecholamines from other organs, e.g., the adrenal medulla. Moreover, pineal melatonin synthesis is not consistently exaggerated by systemic free radical excesses.

In comparison to what is known about Source # 1 melatonin regulation, information about the control of extrapineal/mitochondrial melatonin (Source # 2) production is negligible. It is unknown whether circulating NE influences mitochondrial melatonin synthesis in peripheral organs since melatonin is not usually released from these cells and as a result it would not be reflected in blood melatonin concentrations. This suggests that the mechanisms for the regulation of melatonin stimulation probably differ between these sites.

A recent publication alluded to the possibility that non-visible near infrared radiation (NIR) may be a factor in the regulation of melatonin in peripheral cells. NIR has high penetrability through the skin and into some deeper structures (111). Melatonin is known to be synthesized in dermal and epidermal cells (112, 113) where it has critical functions in the protection of the skin from ROS/RNS induced by ultraviolet radiation exposure or chemical toxins (114). During exposure to the sun, the skin is exposed to both UVR and NIR; these electromagnetic radiations theoretically have contrasting actions in skin cells. Thus, UVR damages dermal and epidermal cells because it generates destructive free radicals while NIR promotes melatonin production in the same cells to combat the associated oxidative stress (115, 116). The beneficial effects of NIR on human health are well recognized, an action that could involve its capacity to promote melatonin production (117). The use of NIR therapy is referred to as photobiomodulation and is widely used for the treatment of a variety of diseases (116).

Melatonin of Source # 2 means this multifunctional molecule is always available, during both the day and at night. Historically, it was presumed by many that the pineal gland was the only or chief source of melatonin. Zhao and colleagues (60) recently proposed that in reality even in those organisms that have a pineal gland less than 5% derives from this organ. If the pineal was the exclusive source of melatonin, which it is obviously not, the functions of this essential agent would be absent during the daily light period when, at least in diurnally-active animals, oxidative stress is highest because of UV exposure, psychological and physical stress, etc. Source # 2 melatonin ensures that it is available during the day when humans are most likely to experience free radical damage. Moreover, if the production of melatonin in peripheral cells is definitively proven to be upregulated by NIR exposure (or any other stimulus) it could be critical in disease prevention and for deferred aging. Finally, in addition to melatonin being available from the pineal and extrapineal tissues, its intake in the diet and contribution made by microbiota may also prove to be significant (116).

**Figure 4:**
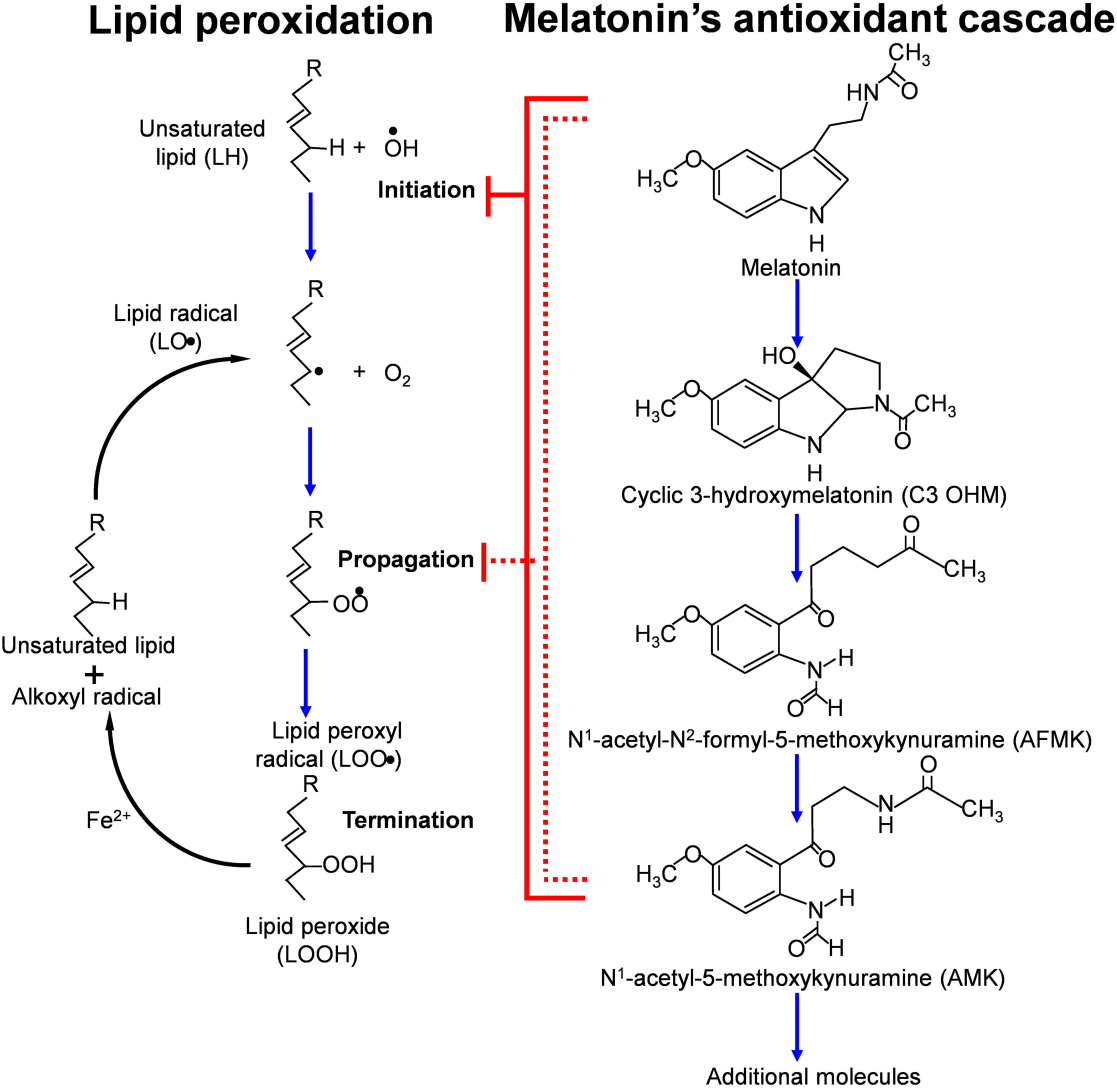
This figure summarizes some of the metabolites that are formed (in what is referred to as the antioxidant cascade) when melatonin functions as a direct free radical scavenger while inhibiting the initiation and propagation of lipid peroxidation. The metabolites of melatonin also function to neutralized ROS/RNS with some of them being more efficient radical scavengers that melatonin itself. Melatonin reduces the peroxidation of unsaturated lipids by scavenging the OH^●^ and other radicals that initiate the breakdown of easily oxidizable lipids. As indicated, the metabolites also interrupt the propagation of lipid peroxidation by neutralizing the lipid peroxyl radical.

While melatonin is well-documented to directly neutralize ROS and RNS, it does not function in this regard without assistance. When melatonin donates and electron to inactivate a radical species, it is transformed into cyclic 3-hydroxymelatonin, which is also a radical scavenger. **Figure 4** illustrates the additional melatonin derivatives that function in scavenging radical species in what is referred to as the melatonin’s antioxidant cascade (118). Some of these derivatives and others are better scavengers than melatonin itself (87, 119, 120). Finally, melatonin chelates transition metals to reduce the formation of the hydroxyl radical during the Fenton reaction or Haber-Weiss reactions (**Figure 4**) (86).

## 4 Discussion

Melatonin is phylogenetically an ancient molecule which, during billions of years of evolution, has been repurposed for a variety of functions. The presently available evidence suggests melatonin first appeared in prokaryotes. There-after, due to an endosymbiotic association that the prokaryotes established with primitive eukaryotes after their internalization, they evolved into mitochondria. Melatonin’s use as an antioxidant possibly was a result of the Great Oxidative Event during which atmospheric oxygen rose profoundly necessitating the need for molecules that were capable of neutralizing toxic oxygen derivatives, i.e., free radicals. High free radical production occurred in primitive animal eukaryotic cells when they began using oxygen as a basis of their metabolism. Additionally, photosynthetic eukaryotes generate oxygen as a byproduct of photo-synthesis that is converted to toxic metabolites under abiotic stress conditions which could damage biomolecules of the photosynthetic apparatus. Over subsequent evolutionary periods to the present day, melatonin retained its ability to detoxify reactive ROS. Moreover, since this function was inherited from engulfed prokaryotes which became mitochondria, the melatonin synthesizing capacity was presumably retained in these organelles to the present day. This ideally positioned melatonin as a radical scavenger since the process of oxidative phosphorylation in mitochondria is the site of the production of the superoxide anion radical, the precursor of all other destructive ROS as well as reactive nitrogen species (RNS) (**Figure 3**).

Many peripheral, perhaps all, cells in tissues of both animal and plant eukaryotic organisms produce melatonin likely as a protection against biomolecular destruction by ROS/RNS as well as other functions. The amount of melatonin synthesized in peripheral cells is presumed to be cell specific and stress de-pendent, i.e., potentially upregulated in animal cells as has been well documented in plants (121). The skin is an example in which proper production of melatonin is linked to protection against solar radiation (10, 113). Mitochondria as the specific site of intracellular melatonin production is generally supported by the published data and is consistent with the evolution of these organelles, which were derived from prokaryotes that presumably had the capacity of melatonin synthesis (**Figure 2**). In addition to the rationale related to evolution of these organelles, compelling arguments for the association of melatonin with mitochondria come from the elegant work of Suofu and colleagues (93) and a publication by He et al. (122). In the latter study, the authors isolated the mitochondria from mouse oocytes, a cell previously shown to synthesize melatonin (123). When these mitochondria were incubated with serotonin as a substrate, they time-dependently generated melatonin; serotonin is a necessary precursor in the melatonin synthetic pathway (110). Conversely, when the oocyte mitochondria were incubated in the absence of serotonin, they failed to produce melatonin. These findings, considered in conjunction with the known contribution of oocyte mitochondria to every cell in vertebrate organisms, support the conclusion that the melatonin synthesizing ability of mitochondria in all cells has been retained to the present day (60). Also, melatonin metabolism within mitochondria can play an important role in their metabolic functions and regulation of cellular phenotype as illustrated in skin cells (124, 125).

Within which mitochondrial compartment melatonin is produced is still under debate. An early immunocytochemical investigation suggested this occurred in the intramembrane space (126). The results of Suofu et al. (93) showed, however, that removal of the outer mitochondrial membrane, using digitoxin, while leaving the inner mitochondrial membrane intact, did not impact the concentration of melatonin in these organelles indicating that it is located in the matrix. Similar to He and colleagues (122), they confirmed that isolated neural mitochondria synthesized melatonin from its deuterated precursor. Despite the currently available data, the possibility that melatonin is produced in other subcellular organelles in addition to mitochondria cannot be precluded (56) and there is the possibility some cells have lost this capacity.

An interaction between Source # 1 and Source # 2 melatonin has been identified and thoroughly investigated by one group of scientists in what is defined as the immune-pineal axis (127). What this group has found is the nocturnal melatonin surge is suppressed by proinflammatory cytokines while simultaneously inducing melatonin synthesis in bone marrow and spleen macrophages (128). This switch in the primary site of melatonin synthesis relies on NF-ĸB activation. These highly mechanistic studies indicate that interleukin-10 (IL-10) levels modulate downstream immune responses which are operative in impacting both Source # 1 (pineal) and Source 2 (extrapineal) melatonin concentrations (89). The alterations in melatonin synthesis are predictably essential in modulating the immune response with the locally-synthesized melatonin by immune-competent cells exerting anti-inflammatory actions at the site of infection (129); the inhibitory actions of melatonin on inflammation have been documented in many studies (130). These intriguing results likely have importance in explaining the efficacy of melatonin as an anti-inflammatory agent and they deserve more intensive investigation.

In the current survey, only the antioxidative actions of peripherally generated melatonin were discussed in detail. Free radical-mediated oxidative damage is a component of many diseases so preventing this damage, which melatonin is highly effective in doing, may be a means by which this important molecule preserves general heath and reduces pathologies. Other disease conditions in which melatonin may have utility as a treatment include cancer, cardiovascular disease, neurodegeneration, sepsis, drug toxicity, and many others. Literature searches will uncover extensive reviews on each of these subjects in reference to melatonin. Melatonin from both Source # 1 and Source # 2 is critical for health maintenance. Moreover, in addition to these two sources of melatonin, it can also be obtained in the diet and supplied by microbiota (131, 132).

## Author contributions

RR: Conceptualization, Data curation, Project administration, Supervision, Validation, Visualization, Writing – original draft. RS: Project administration, Software, Visualization, Writing – review & editing. DT: Data curation, Writing – review & editing. LC: Data curation, Writing – review & editing. Dd: Data curation, Writing – review & editing. AS: Data curation, Funding acquisition, Investigation, Validation, Writing – review & editing. KS: Data curation, Funding acquisition, Investigation, Writing – review & editing. KK: Data curation, Funding acquisition, Investigation, Writing – review & editing.
